# Self-reported prevalence of childhood allergic diseases in three cities of China: a multicenter study

**DOI:** 10.1186/1471-2458-10-551

**Published:** 2010-09-13

**Authors:** Jing Zhao, Juan Bai, Kunling Shen, Li Xiang, Sui Huang, Aihuan Chen, Ying Huang, Jiansheng Wang, Rongwei Ye

**Affiliations:** 1 Capital Institute of Pediatrics in China, Beijing 100020, China; 2 Beijing Children's Hospital attached to Capital Medical University, Beijing 100045, China; 3 GuangZhou Institute of Respiratory Disease, Guangzhou 510120, China; 4 Children's Hospital of Chongqing Medical University, Chongqing 400014, China; 5 Chinese Centre for Disease Control and Prevention, Beijing 100050, China; 6 Peking University Institute of Reproductive and Child Health, Beijing 100191, China

## Abstract

**Background:**

Several studies conducted during the 1990s indicated that childhood allergic diseases were increasing worldwide, but more recent investigations in some Western countries have suggested that the trend is stabilizing or may even be reversing. However, few data are available on the current status of allergic disease prevalence in Chinese children. The aim of the present study was to investigate the prevalence rates of asthma, allergic rhinitis, and eczema in children of three major cities of China, to determine the status of allergic diseases among Chinese children generally, and to evaluate the prevalence of allergic diseases in children of different ages.

**Methods:**

We conducted a cross-sectional survey between October 2008 and May 2009 in three major cities of China (Beijing, Chongqing, and Guangzhou) to evaluate the prevalence rates of childhood allergic diseases including asthma, allergic rhinitis, and eczema, using a questionnaire of the International Study of Asthma and Allergies in Childhood (ISAAC) group. A total of 24,290 children aged 0-14 years were interviewed, using a multi-stage sampling method. To acquire data on children aged 3-14 years, we visited schools and kindergartens. To access children too young to attend school or kindergarten, we extended our survey to community health service centers. Each questionnaire was completed by a parent or guardian of a child after an informed consent form was signed.

**Results:**

Of the 24,290 children in our study, 12,908 (53.14%) were males and 11,382 (46.86%) females; 10,372 (42.70%) were from Beijing, 9,846 (40.53%) from Chongqing, and 4,072 (16.77%) from Guangzhou. Our survey indicated that in Beijing, Chongqing, and Guangzhou, the prevalence rates of asthma were 3.15%, 7.45%, and 2.09%, respectively; the rates of allergic rhinitis were 14.46%, 20.42%, and 7.83%; and the rates of eczema were 20.64%, 10.02%, and 7.22%. The prevalence of allergic diseases varied with age. Asthma was relatively less common both in children aged under 2 years, and in those aged 9 years or more, in each of the three cities. The prevalence of allergic rhinitis was also lower in children younger than 2 years. The prevalence of eczema fell with age.

**Conclusions:**

A marked increase in the prevalence rates of allergic diseases in China (compared with earlier data) was evident. Further studies exploring the precise causes of this increase are warranted.

## Background

Over 25% of the world's population suffer from allergic diseases including asthma, allergic rhinitis, eczema, and drug reactions [1]. Allergic diseases have become one of the top three conditions demanding a major effort toward prevention and control in the 21^st ^century, according to the World Health Organization. In recent years, the prevalence of allergic diseases has increased annually throughout the world [2-6], seriously affecting the quality of life of children, and creating a serious burden on both families and society. If not controlled, allergic diseases can be fatal [7]. Such conditions constitute a challenge for both public health organizations and healthcare providers [8].

In 1997, an ISAAC steering committee studied the prevalence of allergic diseases among children aged 13-14 years in 56 countries, and found that the prevalence of asthma in China was nearly 2.1%, but that in some Western countries including the UK and New Zealand was over 20% [9,10]. Economic development and urbanization in China over the past have dramatically changed both the environmental and lifestyle features of children, but few data are available on the current status of allergic diseases in young Chinese. The aim of the present study was to determine the prevalence rates of these diseases in children from three major cities in China, to provide baseline information for clinicians and health policymakers.

## Methods

### Study population and recruitment of subjects

Our study was conducted in Beijing, Chongqing and Guangzhou, three large cities located in different regions of China. Beijing is in the northern part of the country, and has a relatively low average humidity. Chongqing lies in Southwest China, in a region of high humidity. Guangzhou is in the south of China, and has a subtropical climate.

In each city, two urban districts were randomly selected, and 3-5 schools and kindergartens were chosen at random in each selected district, depending on the population of the city. All children aged 0-14 years in selected schools and kindergartens took part in the survey. To access children who were too young to attend school or kindergarten, we randomly selected two community health service centers that were responsible for managing child community health in each district. A total of 24,290 children was randomly selected, of whom 10,372 were from Beijing, 9,846 from Chongqing, and 4,072 from Guangzhou.

### Questionnaires

An ISAAC questionnaire, a standardized epidemiologic tool used to compare the prevalence of allergic diseases in different regions, was utilized in our survey. The ISAAC questionnaire sought demographic information; and symptoms of asthma, rhinitis, and eczema. All data were acquired during questionnaire-based interviews. To ensure credibility and accuracy, we randomly selected 120 children for re-evaluation of responses 2 weeks after the first evaluation. We thus estimated questionnaire validity and reliability. The Cronbach's α value was 0.82, and the Kappa coefficient of comparison between the two evaluations was 0.81. Asthma, rhinitis, and eczema were considered present if "yes" responses were given to the question: "Has your child ever had asthma, rhinitis, or eczema?" All parents who replied in the affirmative offered details on the ailments suffered by their child. The self-reported prevalence of asthma, rhinitis, and eczema in each city are shown as proportions of the number of affected children, with respect to all questionnaire respondents in any given city.

### Statistical analysis

Data were computer-entered by two researchers using EpiData version 3.1 (double data entry greatly reduces errors). All statistical analyses were performed employing SPSS version 13.0. Measurements are given as means with standard deviations; count data are shown as rates or ratios. The Chi-squared test (χ2) was used to compare differences in the prevalence of asthma and asthma symptoms among cities. In all analyses, P values < 0.05 were regarded as statistically significant.

### Quality-control procedures

Component-specific quality-control procedures were implemented during both the field survey and data analysis to ensure authenticity and reliability of data. To standardize and monitor the quality of data collection and processing, all study personnel received formal training and were certified for all study procedures including face-to-face interviews in kindergartens. A randomly selected sample of 120 completed questionnaires was evaluated to monitor interview quality. All questionnaire data were double-entered to minimize errors, as explained above. Logical checks were performed by statisticians based at the Chinese Centre for Disease Control and Prevention.

### Ethical considerations

Our study was approved by the Ethics Committee of the Capital Institute of Pediatrics in Beijing. A parent or legal guardian of each child gave written informed consent before completing a questionnaire. Any child that appeared to suffer from asthma was investigated further, and, if asthma was diagnosed, the child was prioritized for treatment by an allergist. Parents were offered brochures on asthma treatment and control.

## Results

### Basic information on subjects

Overall, 24,290 children aged 0-14 years took part in the survey. The response rates in Beijing, Chongqing, and Guangzhou were 98.60%, 97.21%, and 90.90%, respectively. Of all children, 12,908 were male and 11,382 female. The gender ratios (male:female) in Beijing, Chongqing, and Guangzhou were 1.11:1, 1.15:1, and 1.13:1, respectively. The mean age of children in the three cities was 7.63 ± 4.04, 8.74 ± 2.72, and 8.39 ± 3.53 years (means ± SDs), respectively. The characteristics of the study subjects are shown in Table 1.

**Table 1:** General information on subject children and response rates in Beijing, Chongqing, and Guangzhou

Characteristic	Beijing		Chongqing		Guangzhou		Total	
	
	n	%	n	%	n	%	n	%
Number of children	10,372	42.70%	9,846	40.53%	4,072	16.77%	24,290	
Response rate	98.60%		97.21%		90.90%		96.62%	
Gender								
Male	5,455	52.59%	5,258	53.40%	2,195	53.90%	12,908	53.14%
Female	4,917	47.41%	4,588	46.60%	1,877	46.10%	11,382	46.86%
Age (mean ± SD)	7.63 ± 4.04		8.74 ± 2.72		8.39 ± 3.53		8.20 ± 3.51	

### Self-reported prevalence of asthma and asthmatic symptoms within the previous 12 months, and self-reported prevalence of rhinitis and eczema

The prevalence of asthma-related symptoms within the previous 12 months was measured by calculating the proportions of subjects who answered "yes" to any of the following questions: "In the last 12 months, has your child had a dry cough at night, apart from a cough associated with a cold or chest infection? Has your child's chest sounded wheezy during or after exercise? Has your child experienced wheezing or whistling in the chest?"

The prevalence of allergic rhinitis in Chongqing was the highest among the three cities studied (20.42%), followed by Beijing and Guangzhou (14.46% and 7.22%, respectively), and the prevalence of asthma in Chongqing (7.45%) was also significantly higher than the rates in Beijing and Guangzhou (3.15% and 2.55%, respectively). However, eczema showed the highest prevalence in Beijing (20.64%), followed by Chongqing and Guangzhou (10.02% and 7.22%, respectively).

Overall, 68.50%, 56.54%, and 68.24% of surveyed asthmatic children in Beijing, Chongqing, and Guangzhou, respectively, had demonstrated asthmatic symptoms (wheezing, cough, or both) within the 12 months prior to the survey, but the prevalence rates of such symptoms in these 12 months did not parallel the prevalence of asthma. The prevalence of wheezing was highest in Beijing (5.54%), whereas exercise-induced asthma and dry cough at night were more prevalent in Guangzhou. Severe eczema symptoms lasting for more than 6 months were present in 1.75%, 2.15%, and 1.38%, respectively, of children in the three cities. In addition, both asthma prevalence and the level of asthma-associated symptoms over the 12 months prior to testing were significantly higher in boys compared to girls, and the same was true when the prevalence rates of rhinitis and eczema were compared. The prevalence of allergic disease and the associated symptoms in the three tested cities are shown in Table 2.

**Table 2:** Prevalence rates of allergic diseases and associated symptoms in Beijing, Chongqing, and Guangzhou

Characteristic	Beijing		Chongqing		Guangzhou		χ2	P
	
	n	%	n	%	n	%		
**Asthma**								
Asthma at any time	324	3.15%	710	7.45%	115	2.55%	309.16	< 0.001
Male	223	4.09%	432	8.22%	30	1.37%	172.49	< 0.001
Female	101	2.05%	278	6.06%	85	4.53%	98.54	< 0.001
**Symptoms in last 12 months**								
Wheeze	575	5.54%	343	3.48%	160	3.93%	20,074.52	< 0.001
Exercise-induced wheeze	705	6.80%	703	7.14%	344	8.45%	17,618.17	< 0.001
Dry cough at night	903	8.71%	852	8.65%	358	8.79%	16,381.75	< 0.001
**Rhinitis**								
Rhinitis at any time	1,500	14.46%	2,011	20.42%	294	7.22%	11,099.48	< 0.001
Male	897	16.44%	1,161	22.08%	196	8.93%	4,543.83	< 0.001
Female	603	12.26%	850	18.53%	123	6.55%	5,775.32	< 0.001
**Eczema**								
Eczema at any time	2,141	20.64%	1,085	10.02%	294	7.22%	12,537.00	< 0.001
Eczema >6 months in duration	181	1.75%	212	2.15%	56	1.38%	22,463.97	< 0.001
Male	1,153	21.13%	596	11.34%	165	7.52%	6,512.16	< 0.001
Female	988	20.09%	489	10.66%	129	6.87%	5,958.25	< 0.001

### Coexistence of various allergic diseases

Of all asthmatic children, 49.54%, 50.14%, and 34.83% also had allergic rhinitis in Beijing, Chongqing, and Guangzhou, respectively. The proportions of asthmatic children with eczema in these cities were 45.23%, 32.29%, and 19.10%, respectively. The percentages of asthmatic children with both allergic rhinitis and eczema were 23.08%, 21.25%, and 10.11%, respectively, in the three cities. Of children with allergic rhinitis, 10.73%, 18.30%, and 10.20% suffered from asthma. The extent of coexistence of allergic diseases is shown in Table 3.

**Table 3:** Coexistence of allergic diseases in children aged 0-14 years in Beijing, Chongqing, and Guangzhou (number and %)

Coexistence	Beijing		Chongqing		Guangzhou		χ2	P
			
	n	%	n	%	n	%		
Rhinitis in asthmatic children	161	49.54%	368	50.14%	31	34.83%	22,021.76	<0.001
Eczema in asthmatic children	147	45.23%	237	32.29%	17	19.10%	22,655.07	<0.001
Rhinitis and eczema in asthmatic children	75	23.08%	156	21.25%	9	10.11%	23,304.58	<0.001

### Prevalence of allergic diseases in children of different ages

The prevalence of allergic diseases varied with age. The prevalence of asthma was highest in children aged 2-9 years, whereas asthma prevalence rates in younger and older children were lower, in all three cities. The prevalence of allergic rhinitis was also lowest in children under the age of 2 years, and highest in children aged 6-11 years. Eczema was most common in children under 5 years of age, and decreased with age. However, the prevalence of eczema in children aged 13-14 years exhibited a small rebound in Guangzhou. The prevalence of allergic diseases in children of different ages is shown in Table 4.

**Table 4:** Prevalence of allergic diseases in children of different ages

Age	Beijing			Chongqing			Guangzhou		
	
	Asthma	Rhinitis	Eczema	Asthma	Rhinitis	Eczema	Asthma	Rhinitis	Eczema
0-1 years	0.55	1.78	31.05	0	0	42.86	0	1.24	13.58
1-2 years	1.76	4.76	29.98	0	15.63	6.25	3.73	4.48	13.43
2-3 years	4.47	10.68	30.93	11.26	9.91	22.97	4.07	6.67	17.78
3-4 years	4.33	15.43	31.29	9.70	18.99	20.68	2.18	8.36	13.45
4-5 years	5.00	18.23	23.39	12.29	16.57	17.14	3.92	9.48	10.13
5-6 years	4.86	18.15	24.15	8.30	18.96	13.67	3.32	9.54	8.71
6-7 years	3.93	19.97	20.60	8.57	20.95	13.26	2.09	10.99	5.76
7-8 years	3.70	19.98	22.22	9.35	21.95	13.05	2.62	9.69	7.07
8-9 years	4.46	20.89	18.85	8.14	21.61	11.67	2.88	8.39	4.08
9-10 years	3.76	19.25	16.64	6.15	20.29	7.25	0.95	5.92	2.84
10-11 years	3.51	19.56	16.42	4.80	18.59	4.65	0.92	8.90	3.99
11-12 years	1.08	11.11	10.27	4.66	20.55	5.89	1.01	5.6	4.05
12-13 years	1.72	10.83	8.43	4.42	22.25	5.18	0.93	7.89	4.41
13-14 years	1.48	14.05	7.39	3.83	25.2	9.69	0.98	9.80	8.82
Total	3.15	14.46	20.64	7.45	20.42	10.02	2.55	7.22	7.22

## Discussion

Allergic diseases are very common in children worldwide [9]. A survey by the World Allergy Organization, covering 1.2 billion children, found that 250 million suffered from allergic diseases [11]. Such conditions may limit the ability of a child to play, to learn, and to sleep. Allergic conditions require potentially complex and expensive therapeutic intervention, and are thus associated with both direct and indirect costs (*e.g.*, missed school and work days) [12-14]. During the 1990s, the prevalence of childhood allergic diseases increased considerably in several countries [15-18]. Such diseases are a major burden on affected children and their families, and are becoming a serious challenge to public health organizations and healthcare providers [8].

ISAAC conducted a multiphase cross-sectional study, beginning in 1991, to facilitate research into asthma, allergic rhinitis, and eczema, and to allow comparisons of the prevalence of allergic diseases between populations in different regions. ISAAC developed a standardized epidemiologic tool to measure the prevalence of various childhood allergic conditions [19,20]. In 1997, the ISAAC steering committee investigated the prevalence rates of allergic diseases in children aged 13-14 years in 58 countries, and found that asthma prevalence differed 20- to 60-fold across countries [9]. Among the countries in the ISAAC study, the United Kingdom, Australia, New Zealand, and some other Western countries showed prevalence rates of asthma of more than 20%, and the rates of individuals who complained of symptoms of asthma in the 12 months prior to analysis attained 30% [10]; these rates were higher than the corresponding rates in other countries. In an earlier survey, the prevalence rates of asthma and rhinitis in children in Mainland China were 2.1% and 9.1%, respectively [10]. However, we found that the prevalence rates of asthma in Beijing, Chongqing, and Guangzhou were 3.15%, 7.45%, and 2.55% respectively; and the rates of rhinitis were 14.46%, 20.42%, and 7.22% respectively. In 1997, the prevalence rates of wheeze/exercise-induced wheeze and dry cough among children, at any time in the past year, were 1.4%, 6.6%, and 7.1%, respectively, in Mainland China. In our present survey of children in Beijing, Chongqing, and Guangzhou, the prevalence rates of wheeze were 5.54%, 3.48%, and 3.93%, respectively; the rates of exercise-induced wheeze were 6.80%, 7.14%, and 8.45%, respectively; and the rates of dry cough were 8.71%, 8.65%, and 8.79%, respectively.

Many factors, including genetic features, environmental influences, and social status, affect the prevalence of allergic diseases, making it very difficult to offer a comprehensive explanation of observed trends. Previous studies suggested that the observed worldwide increase in the prevalence of allergic diseases might be linked to lifestyle changes associated with urbanization [21]. For example, a population-based study in Mongolia found that the prevalence of allergic sensitization increased significantly as population density increased, being 13.6% in villages, 25.3% in rural towns, and 31.0% in a city [22] In the past few decades, the economy of China has gone through a period of unprecedented rapid development, leading to great changes in lifestyle, development of urbanization, and increasing "Westernization" of environmental factors [23]. Changes in maternal diet, smaller family size, the occurrence of fewer infections during infancy, lowered exposure to rural environments, and improvements in sanitation may partly explain the observed trend; however, further research is needed to achieve a full understanding.

In our survey, children in Chongqing showed significantly higher prevalence rates of asthma and allergic rhinitis compared to children living in Beijing or Guangzhou, consistent with the findings of a previous national epidemiological survey, conducted in 2000, of asthma prevalence in children aged 0-14 years [22]. This geographic variation may be part-explained by variations in environmental and climate factors [24]. Chongqing is in southwest China, where the humidity is relatively high, thus favoring the growth of mites that cause allergic disease [25]. Other social and environmental factors should be examined in future studies. We also found that the prevalence of asthma in males was significantly higher than in females in each city, indicating a gender-based difference in the prevalence of allergic diseases in children, in line with data from previous studies. It is probable that both hormonal changes with age, and genetic susceptibility, contribute to such differences [26]. Besides, boys, who tend to engage in more outdoor activities, are better protected against development of allergic diseases [27]. As the age of children increased, the prevalence rates of asthma, allergic rhinitis, and eczema showed different trends. The prevalence of asthma was low in both very young and older children. Allergic rhinitis was relatively uncommon in children aged less than 5 years. The prevalence of eczema gradually decreased as children grew. Such variations suggest that healthcare and governmental authorities may need to construct age-dependent preventative strategies to control allergic diseases in children.

In recent years, some scholars have proposed that asthma and allergic rhinitis share a pathological mechanism, and that the conditions are different (but closely allied) clinical manifestations of chronic inflammation of the upper and lower respiratory tracts [28-59]. In some Western counties, 30-90% of patients with asthma also have allergic rhinitis [31,32], and the latter condition has been proposed to be an independent risk factor for asthma [30,32]. In our survey, 49.54%, 50.14%, and 34.83% of asthmatic children suffered also from allergic rhinitis in Beijing, Chongqing, and Guangzhou, respectively. This observation is in line with the above suggestion. In addition, the proportions of asthmatic children who also had eczema, and those of asthmatic children who suffered from both allergic rhinitis and eczema, were high in all three cities, indicating that allergic diseases often occur in combination.

A common shortcoming of questionnaire-based studies is selection bias. In the present study, our subjects included children in kindergartens, primary and secondary schools, and those under kindergarten age. Sampling method is also a potential source of bias. In the present study, we measured child numbers in each age group, and randomly selected subjects with reference to these numbers, to ensure that our data was representative. One potential limitation of the survey is that rural-dwelling children were not included. Also, although we conducted our work in three widely separated cities--Beijing, Chongqing, and Guangzhou--differences in the risk factors for asthma/allergic rhinitis/eczema, and the reasons for variations in disease prevalence among the three cities, were not further investigated.

## Conclusions

We used an ISAAC questionnaire to study the prevalence rates of allergic diseases in children aged 0-14 years living in three major cities in China. We found that the prevalence rates of such diseases were significantly higher than in previous surveys, and that the gap that previously existed in the prevalence of allergic diseases between China and Western countries is gradually narrowing. Environmental and lifestyle changes may have contributed to this trend. Further studies are necessary to discern the precise reasons explaining the observed trend. We also found that the prevalence rates of allergic diseases were relatively high in very young children. In addition, all of asthma, allergic rhinitis, and eczema, the principal allergic diseases in children, often coexist, and may thus mutually interact to increase the severity of each individual condition.

## Competing interests

The authors declare that they have no competing interests.

## Authors' contributions

JZ and JB participated in study design and performed statistical analysis. LX, KLS, SH, AHC, and YH administered questionnaires in Beijing, Guangzhou, and Chongqing. JSW and RWT conducted quality-control work, including training of all investors. All authors have read and approved of the final manuscript.

## Pre-publication history

The pre-publication history for this paper can be accessed here:

http://www.biomedcentral.com/1471-2458/10/551/prepub
